# Knockout of *tusA* confers cationic antimicrobial resistance via *fur* and *omp* genes in *Escherichia coli*

**DOI:** 10.1128/jb.00103-26

**Published:** 2026-04-22

**Authors:** Kazuya Ishikawa, Kiho Nakata, Koichiro Yamada, Mio Uneme, Kazuyuki Furuta, Chikara Kaito

**Affiliations:** 1 Graduate School of Medicine, Dentistry and Pharmaceutical Sciences, Okayama University 12997, Okayama, Japan; University of Notre Dame, Notre Dame, Indiana, USA

**Keywords:** *tusA*, *fur*, *Escherichia coli*, cationic antimicrobial agents

## Abstract

tRNA 2-thiouridine synthesizing protein A (TusA), a sulfur-carrier protein, plays a crucial role in tRNA sulfur modification. Several studies have reported that *tusA* deficiency affects iron–sulfur (Fe–S) homeostasis in addition to tRNA sulfur modification, resulting in pleiotropic phenotypes. In this study, we analyzed the phenotype of *tusA*-deficient *Escherichia coli* and its underlying mechanisms. Although the Keio *tusA* knockout strain showed increased swimming motility and flagellar biosynthesis, these phenotypes were not restored by *tusA* complementation. Genome resequencing of the original Keio *tusA* knockout strain identified an unintended secondary mutation in *lrhA*, a transcriptional regulator of flagellar and chemotaxis genes. This secondary mutation impaired *lrhA* function, contributing to enhanced flagellar synthesis and swimming motility, as well as altered global gene expression. A *tusA* knockout strain in which the secondary mutation was corrected (Δ*tusA*) exhibited reduced swimming motility compared with the wild-type strain, despite showing no abnormalities in flagellar formation. Furthermore, Δ*tusA* displayed increased resistance to cationic antibacterial agents, including cetyltrimethylammonium bromide, cetylpyridinium chloride, and protamine sulfate. The reduced motility caused by *tusA* deletion was observed independently of Fur, a global regulator of iron homeostasis, whereas resistance to cationic antibacterial agents was abolished in the *fur* knockout background. In addition, altered expression of outer membrane protein (*omp*) genes was observed in Δ*tusA*, and deletion of these *omp* genes abolished resistance to cationic antibacterial agents. Together, these results indicate that, by disentangling the phenotypic effects of *tusA* deletion from those of the unintended secondary mutation, loss of *tusA* confers resistance to cationic antibacterial agents through a Fur-dependent and Omp-dependent mechanism.

**IMPORTANCE:**

tRNA 2-thiouridine synthesizing protein A (TusA) is a sulfur-carrier protein involved in tRNA thiolation and iron–sulfur (Fe–S) cluster homeostasis. Accordingly, deletion of *tusA* results in pleiotropic phenotypes. The Keio *tusA* knockout strain is widely used to study *tusA* function; however, resequencing revealed a secondary mutation in *lrhA*, a transcriptional regulator of flagellar biosynthesis and chemotaxis. This *lrhA* mutation affected flagellar formation, swimming motility, and gene expression in the Keio *tusA* knockout strain. We further demonstrated that a *tusA* knockout strain with repaired *lrhA* exhibited reduced swimming motility and increased resistance to cationic antimicrobial agents. Our findings highlight the impact of the secondary mutation in the widely used Keio *tusA* knockout strain and provide insights into the functions of *tusA*.

## INTRODUCTION

tRNA 2-thiouridine synthesizing protein A (TusA) is a sulfur-carrier protein that participates in molybdenum cofactor biosynthesis and tRNA thiolation (1, 2). TusA receives sulfur from cysteine via the cysteine desulfurase IscS and subsequently transfers it to the TusBCD complex, TusE, and MnmA. This sulfur relay ultimately leads to thiolation of uridine at the wobble position (U34) of tRNAs for Lys, Gln, and Glu, resulting in the formation of 5-methylaminomethyl-2-thiouridine (mnm5s2U) (3, 4). This modification stabilizes tRNA structure and enhances the accuracy and efficiency of protein translation (5).

Iron–sulfur (Fe–S) clusters are essential cofactors for numerous enzymes, particularly those involved in electron transport and redox reactions (6). The two major types of Fe–S clusters, [2Fe–2S] and [4Fe–4S], are composed of iron and inorganic sulfide coordinated by cysteine residues in proteins. Fe–S clusters are primarily assembled by the iron sulfur clusters (ISC) and sulfur formation (SUF) systems, which mediate sulfur mobilization, iron delivery, and scaffold-dependent cluster assembly. Because Fe–S clusters are critical for cellular functions, their biogenesis and maintenance are tightly regulated as part of Fe–S homeostasis (7, 8).

TusA-deficient (Δ*tusA*) *Escherichia coli* exhibits multiple phenotypes, including impaired cell growth (9, 10), phage resistance associated with altered frameshift efficiency (10), and reduced translation efficiency of transcriptional regulators (11, 12). In addition, deletion of *tusA* has been reported to decrease the levels of [4Fe–4S] clusters in *E. coli*, thereby disrupting Fe–S homeostasis (12). Two models have been proposed to explain this phenomenon. The first model posits that loss of tRNA thiolation caused by *tusA* deletion increases the availability of sulfur for Fe–S cluster biogenesis, because both tRNA thiolation and Fe–S cluster assembly utilize cysteine as a sulfur source and the cysteine desulfurase IscS as a central sulfur donor (13). The second model suggests that *tusA* deficiency reduces the translation efficiency of the transcriptional regulator Fur, which controls iron homeostasis and Fe–S cluster biogenesis (12). Consistent with these models, microarray analyses have shown that, in Δ*tusA*, the expression of genes involved in Fe–S cluster biogenesis and in the regulon of the Fe–S cluster-containing transcription factor Fnr is altered (13). The pleiotropic phenotypes of Δ*tusA* are therefore likely attributable to the roles of TusA in the translation of multiple transcriptional regulators and in the maintenance of Fe–S homeostasis. However, the mechanistic links between each phenotype of Δ*tusA* and its underlying molecular basis remain poorly understood.

The Keio collection is a comprehensive *Escherichia coli* knockout library consisting of 3,985 single-gene deletion mutants (14) and is widely used as a powerful genetic resource for functional studies. In this collection, nearly the entire open reading frame of each target gene is precisely replaced with an antibiotic resistance cassette that can be readily excised, making the system well suited for genetic analyses. However, several Keio deletion strains have been reported to harbor unintended secondary mutations (15), representing an important caveat for their use. The *tusA* knockout strain from the Keio collection (JW3435; hereafter referred to as Δ*tusA*-k) has likewise been employed in numerous previous studies (10–13, 16).

In this study, we characterized the phenotypes of Δ*tusA*-k and investigated their underlying molecular mechanisms. During this analysis, we observed several phenotypes in Δ*tusA*-k that were not complemented by the introduction of *tusA*. Suspecting the presence of a secondary mutation, we performed whole-genome resequencing and identified a nonsense mutation in *lrhA*, a transcriptional regulator that controls the expression of genes involved in flagellar biosynthesis and chemotaxis. Furthermore, by repairing the *lrhA* mutation to the wild-type (WT) allele, we demonstrated that deletion of *tusA* results in reduced swimming motility and increased resistance to cationic antimicrobial agents.

## RESULTS

### The Keio Δ*tusA* strain exhibits increased swimming motility and flagellar formation that are not complemented by *tusA* introduction

**Fig 1:**
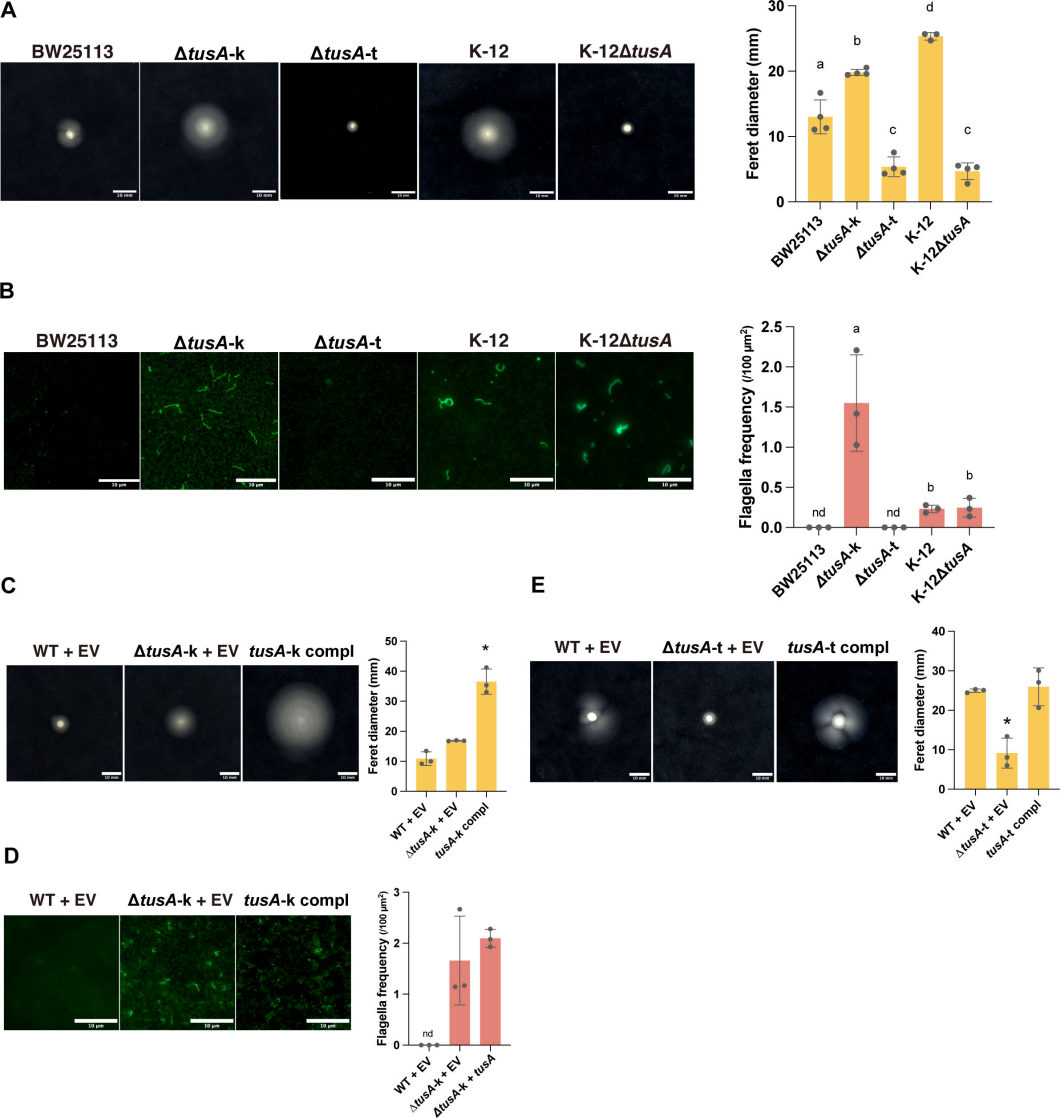
A *tusA* knockout mutant from the Keio collection exhibits enhanced swimming motility and increased flagellation. (**A**) Swimming motility of BW25113, K-12, and their *tusA*-deficient mutants (Δ*tusA*-k, a *tusA* knockout derived from the Keio collection; Δ*tusA*-t and K-12 Δ*tusA*, *tusA* knockouts generated by phage transduction using Δ*tusA*-k as the donor and BW25113 or K-12 as the recipients, respectively). Soft agar plates were incubated at 37°C for 19 h. Scale bars, 10 mm. Quantification of swimming halo diameters is shown. Data are presented as mean ± SD; different letters indicate significant differences (*n* = 4, *P* < 0.01, Tukey’s multiple comparisons test). (**B**) Immunofluorescence staining using an anti-flagellin antibody. Bacterial cultures of the same strains as in panel **A** were visualized by fluorescence microscopy. Scale bar, 10 µm. Quantification of flagellar frequency per area is shown. Data are presented as mean ± SD; different letters indicate significant differences (*n* = 3, *P* < 0.01, Tukey’s multiple comparisons test). (**C**) Swimming motility of BW25113 (WT) and Δ*tusA*-k harboring pCA24N (WT + EV, Δ*tusA*-k + EV) or pCA24N-tusA (Δ*tusA*-k compl). Experimental conditions and quantification were the same as in panel **A**. An asterisk indicates significant difference from the others. (**D**) Immunofluorescence staining of WT + EV, Δ*tusA*-k + EV, and Δ*tusA*-k compl using an anti-flagellin antibody. Experimental conditions and quantification were the same as in panel **B**. An asterisk indicates significant difference from the others. (**E**) Swimming motility of BW25113 and Δ*tusA*-t harboring pCA24N (WT + EV, Δ*tusA*-t + EV) or pCA24N-tusA (Δ*tusA*-t compl). Experimental conditions and quantification were the same as in panel **A**, except that plates were incubated for 24 h due to the low swimming motility of the strains.

A previous study reported enhanced accumulation of flagellin in the *tusA* knockout strain from the Keio collection (Δ*tusA*-k) (13). Based on this observation, we hypothesized that Δ*tusA*-k would exhibit altered swimming motility compared with its parental strain, BW25113. As expected, Δ*tusA*-k displayed significantly greater swimming motility than the wild-type strain (Fig. 1A). To determine whether this phenotype was directly caused by *tusA* deletion, we constructed a transductant strain (Δ*tusA*-t) by transferring the chromosomal region containing the *tusA* deletion from Δ*tusA*-k into the wild-type strain via phage transduction. In contrast to Δ*tusA*-k, Δ*tusA*-t exhibited significantly reduced swimming motility compared with the wild type (Fig. 1A).

Because BW25113 is a K-12 derivative with relatively low intrinsic swimming motility (17), we additionally generated a *tusA* deletion mutant in the original K-12 genetic background. The K-12 Δ*tusA* strain also showed significantly reduced swimming motility compared with its parental strain (Fig. 1A). Taken together, these results indicate that deletion of *tusA* itself reduces swimming motility, whereas the increased motility observed in Δ*tusA*-k is attributable to the presence of a secondary mutation.

Next, we examined flagellar formation in each strain by immunofluorescence microscopy. BW25113 produced very few flagella, whereas Δ*tusA*-k formed abundant flagella (Fig. 1B). In contrast, Δ*tusA*-t showed minimal flagellar formation, comparable to that of the parental strain (Fig. 1B). In the original K-12 genetic background, both the parental strain and the Δ*tusA* mutant exhibited similar levels of flagella (Fig. 1B). These results suggest that deletion of *tusA* has little to no effect on flagellar formation, and that the enhanced flagellation observed in Δ*tusA*-k is attributable to a secondary mutation.

To determine whether the enhanced swimming motility and flagellar formation of Δ*tusA*-k were caused by a secondary mutation, we introduced *tusA* into both Δ*tusA*-k and Δ*tusA*-t. In Δ*tusA*-k, complementation with *tusA* did not restore the phenotypes; instead, swimming motility was further increased (Fig. 1C). Flagellar formation was not significantly affected by *tusA* complementation (Fig. 1D). In contrast, the introduction of *tusA* into Δ*tusA*-t restored swimming motility to near wild-type levels (Fig. 1E). These results indicate that the enhanced swimming motility and flagellar formation observed in Δ*tusA*-k are caused by a secondary mutation rather than by deletion of *tusA*. We also examined the growth curves of Δ*tusA*-k and Δ*tusA*-t in LB medium. In both mutants, the growth defect was rescued by *tusA* complementation (Fig. S1), indicating that the basal growth impairment of Δ*tusA*-k and Δ*tusA*-t is attributable to the loss of *tusA*.

### The Keio *tusA* knockout strain carries a nonsense mutation in *lrhA*

**Fig 2:**
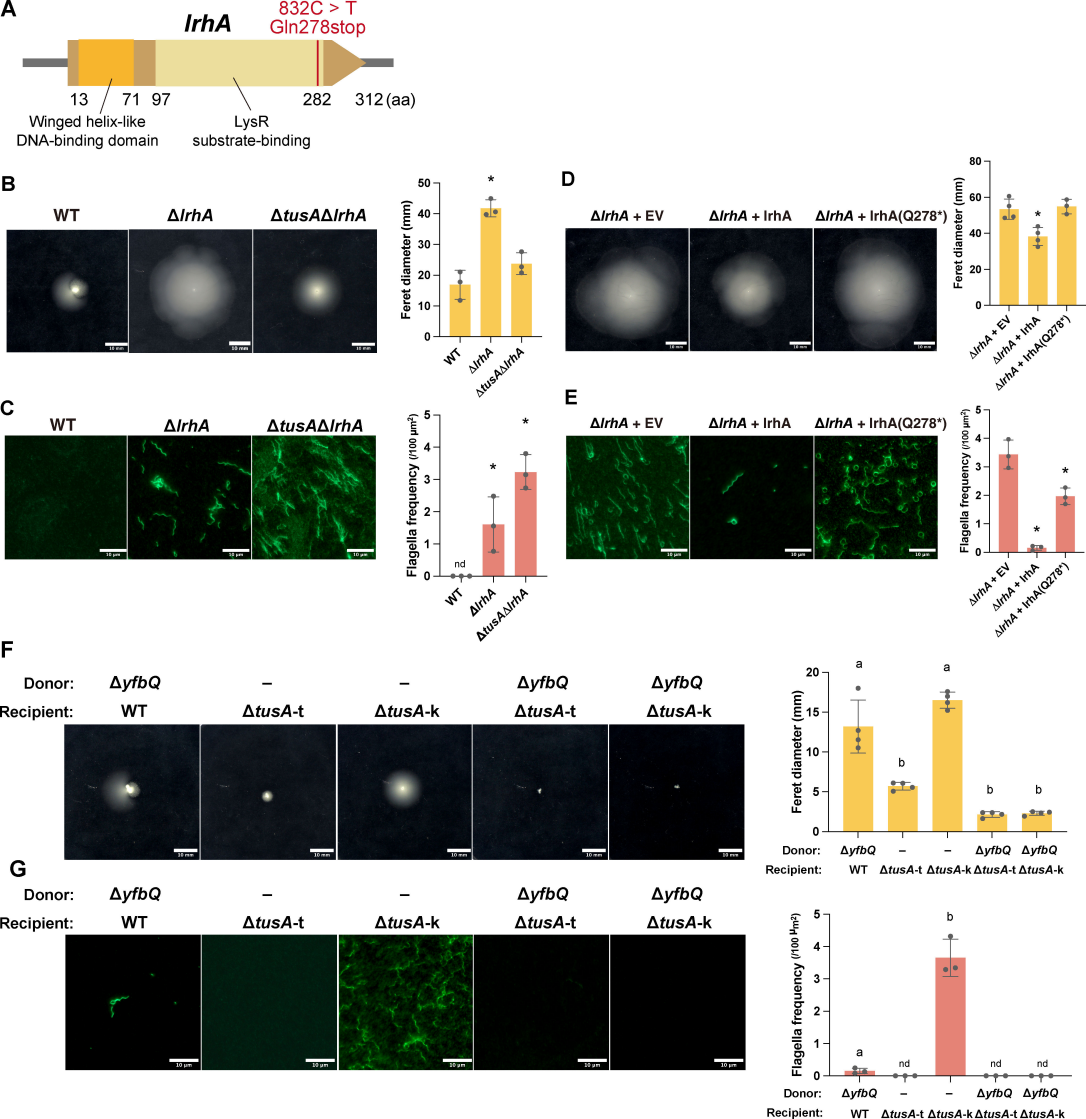
The Keio *tusA* knockout strain (Δ*tusA*-k) carries a nonsense mutation in *lrhA*. (**A**) Schematic illustration of the secondary mutation in *lrhA* in Δ*tusA*-k. Protein domains were annotated based on the InterPro database. (**B**) Swimming motility of BW25113 (WT), Δ*lrhA*, and Δ*tusA* Δ*lrhA*. Soft agar was stab-inoculated and incubated at 37°C for 24 h. Scale bars, 10 mm. Quantification of swimming halo diameters is shown. Data are presented as mean ± SD; asterisks indicate significant differences from WT (*n* = 3, *P* < 0.01, Tukey’s multiple comparisons test). (**C**) Immunofluorescence staining using an anti-flagellin antibody. Bacterial cultures of the same strains as in panel (**B**) were visualized by fluorescence microscopy. Scale bars, 10 µm. Quantification of flagellar frequency per area is shown. Data are presented as mean ± SD; asterisks indicate significant differences from WT (*n* = 3, *P* < 0.01, Tukey’s multiple comparisons test). (**D**) Swimming motility of Δ*lrhA* harboring the empty vector (Δ*lrhA* + EV), a plasmid expressing wild-type *lrhA* (Δ*lrhA + lrhA*), or a plasmid expressing *lrhA*(Q278*) (Δ*lrhA + lrhA*[Q278*]). Experimental conditions and quantification were the same as in panel **B**, except that plates were incubated for 19 h. (**E**) Immunofluorescence staining of Δ*lrhA* + EV, Δ*lrhA + lrhA*, and Δ*lrhA + lrhA*(Q278*) using an anti-flagellin antibody. Experimental conditions and quantification were the same as in panel **C**. (**F and G**) Swimming motility (**F**) and immunofluorescence staining (**G**) of Δ*tusA*-t, Δ*tusA*-k, and transductants generated by P1 phage transduction using Δ*yfbQ* as the donor and BW25113, Δ*tusA*-t, or Δ*tusA*-k as the recipients. Experimental conditions and quantification were the same as in panels **B** and **C**, respectively.

To identify the mutation responsible for the enhanced swimming motility and flagellar formation in Δ*tusA*-k, we performed whole-genome resequencing. Comparison with Δ*tusA*-t revealed a single-nucleotide substitution: a C-to-T transition at nucleotide position 832 of *lrhA*, which encodes a transcriptional regulator that represses genes involved in flagellar biosynthesis and chemotaxis (18) (Fig. 2A). This single-nucleotide polymorphism converts the glutamine codon at position 278 into a stop codon, resulting in a nonsense mutation that truncates the C-terminal region of LrhA. To exclude the possibility that this mutation arose during strain manipulation in our laboratory, we re-obtained the Δ*tusA*-k strain and sequenced the *lrhA* locus, which confirmed the presence of the same mutation. Because this mutation was predicted to account for the increased swimming motility and flagellar formation observed in Δ*tusA*-k, we next examined the motility of an *lrhA* deletion strain (Δ*lrhA*). Δ*lrhA* exhibited significantly higher swimming motility than the parental strain (Fig. 2B). Furthermore, a double-knockout strain (Δ*tusA* Δ*lrhA*) displayed significantly lower swimming motility than Δ*lrhA*, at a level comparable to that of Δ*tusA*-k (Fig. 2B).

Immunofluorescence analysis revealed that both Δ*lrhA* and the Δ*tusA* Δ*lrhA* double mutants formed abundant flagella (Fig. 2C). When either wild-type *lrhA* or the *lrhA* allele from Δ*tusA*-k [*lrhA*(Q278*)] was introduced into Δ*lrhA*, expression of wild-type *lrhA* significantly reduced swimming motility, whereas *lrhA*(Q278*) showed only minimal effects (Fig. 2D). Consistently, the introduction of wild-type *lrhA* markedly decreased flagellar formation, whereas *lrhA*(Q278*) resulted in only a modest reduction (Fig. 2E). Together, these results indicate that LrhA acts as a repressor of flagellar formation and swimming motility, and that the LrhA(Q278*) variant has largely lost its regulatory function.

To determine whether the *lrhA*(Q278*) mutation in Δ*tusA*-k is responsible for the altered swimming motility, we replaced the mutant *lrhA* allele with the wild-type allele by P1 phage-mediated transduction using a Keio Δ*yfbQ* strain, which carries a deletion in a gene located in close proximity to *lrhA*, as the donor. The resulting transductant exhibited reduced swimming motility compared with the parental Δ*tusA*-k strain (Fig. 2F). Consistently, immunofluorescence analysis showed that the enhanced flagellar formation observed in the parental Δ*tusA*-k strain was no longer detectable in the transductant (Fig. 2G). These results demonstrate that both the increased swimming motility and the enhanced flagellar formation of Δ*tusA*-k are attributable to the *lrhA*(Q278*) nonsense mutation.

Both *rpoS* and *fur* have been reported to be functionally linked to *tusA* (11, 12), and deletion of either gene is known to affect swimming motility (19, 20). To elucidate the mechanism underlying the reduced swimming motility of Δ*tusA*-t, we constructed single knockout mutants (Δ*fur* and Δ*rpoS*) and double mutants lacking *tusA* (Δ*tusA* Δ*fur* and Δ*tusA* Δ*rpoS*) and examined their swimming motility. Both Δ*fur* and Δ*rpoS* exhibited markedly increased swimming motility compared with the wild type (Fig. S2). In the *tusA*-deleted background, however, the enhanced motility conferred by *fur* deletion was abolished, whereas the Δ*tusA* Δ*rpoS* double mutant retained an intermediate level of motility between that of Δ*rpoS* and the wild type. These results indicate that the increased swimming motility caused by *fur* deletion depends on the presence of *tusA*, whereas the enhanced motility mediated by *rpoS* deletion occurs largely independently of *tusA*.

### Transcriptome analysis of Δ*tusA*-k and Δ*tusA*-t

**Fig 3:**
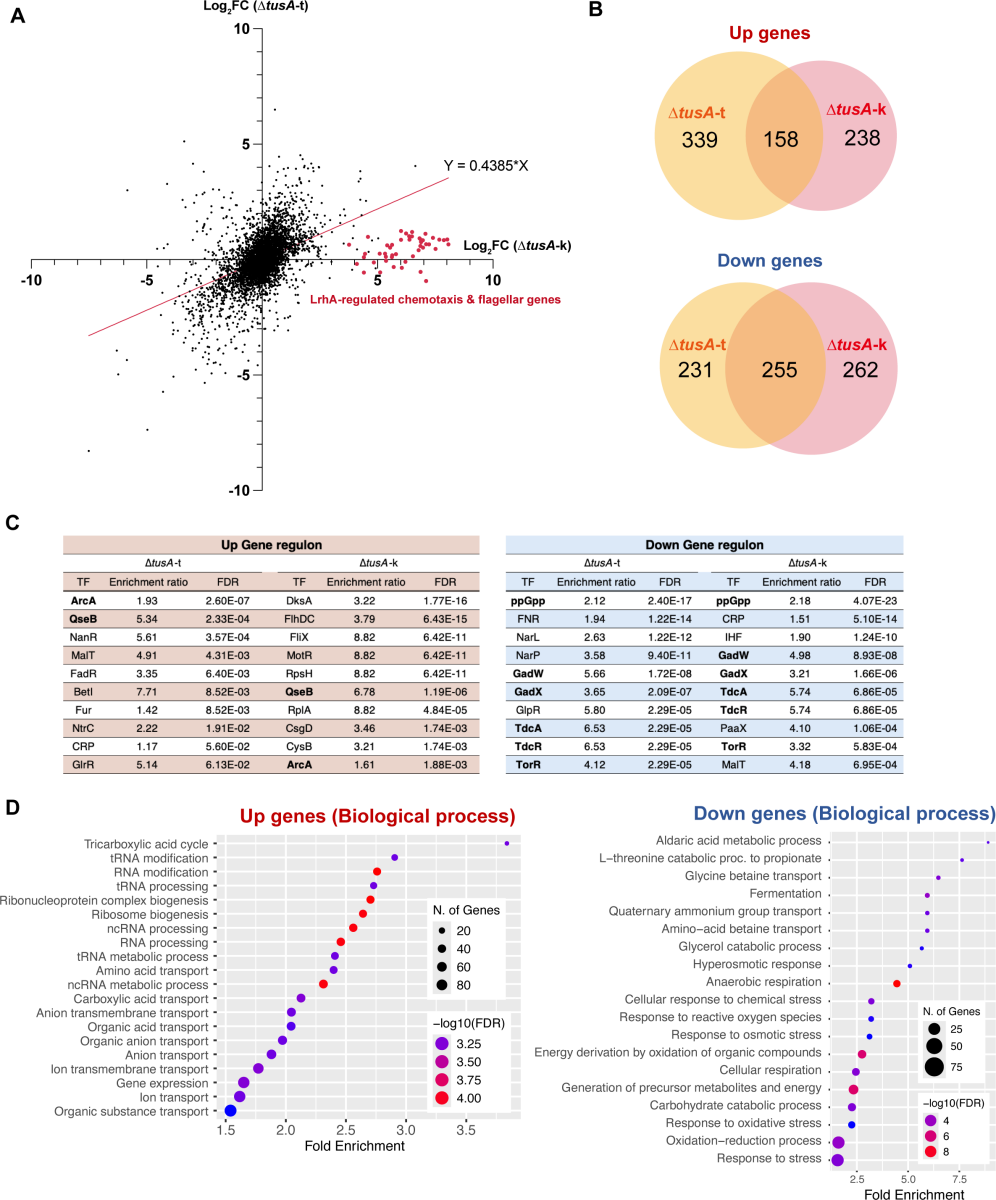
Comparison of RNA expression between ∆*tusA*-t and ∆*tusA*-k. (**A**) Fold-change plot of gene expression in ∆*tusA*-t or ∆*tusA*-k relative to BW25113. The *x*-axis represents the Log2 fold change (FC) of each gene in ∆*tusA*-k relative to BW25113. The *y*-axis represents the Log2 fold change (FC) of each gene in ∆*tusA*-t relative to BW25113. The red plots indicate flagella-related and chemotaxis-related genes whose expression is regulated by *lrhA*. The red line is the linear regression line. (**B**) Venn diagram of upregulated and downregulated genes in ∆*tusA*-t and ∆*tusA*-k. Genes whose expression varied by more than twofold and had a *P* value of 0.05 or less were counted. (**C**) Regulon enrichment analysis. The transcription factor regulons enriched in upregulated or downregulated genes were analyzed. The top 10 transcription factors with false discovery rate (FDR) are shown. Bold indicates transcription factors that were in the top 10 for both ∆*tusA*-t and ∆*tusA*-k. (**D**) GO enrichment analysis of upregulated or downregulated genes in ∆*tusA*-t.

To evaluate the effects of the *lrhA*(Q278*) mutation and *tusA* deletion on genome-wide gene expression, we performed RNA sequencing (RNA-seq) of Δ*tusA*-k and Δ*tusA*-t (Fig. S1). When the fold changes in gene expression for each gene relative to the wild type were plotted (Fig. 3A and Table S1), the correlation coefficient between Δ*tusA*-k and Δ*tusA*-t was 0.43, indicating substantial differences between the two transcriptomes(21). Notably, flagellar and chemotaxis-related genes that were strongly upregulated in Δ*tusA*-k showed little or no change in Δ*tusA*-t, indicating a pronounced effect of the *lrhA*(Q278*) mutation present in Δ*tusA*-k (Fig. 3A).

Next, we compared differentially expressed genes showing at least a twofold change relative to the wild type between Δ*tusA*-k and Δ*tusA*-t. A total of 158 genes were commonly upregulated in both Δ*tusA*-k and Δ*tusA*-t, whereas 238 and 339 genes were uniquely upregulated in Δ*tusA*-k and Δ*tusA*-t, respectively (Fig. 3B). For downregulated genes, 255 genes were commonly downregulated in both strains, while 262 and 231 genes were uniquely downregulated in Δ*tusA*-k and Δ*tusA*-t, respectively (Fig. 3B). The number of commonly downregulated genes exceeded that of commonly upregulated genes, which may reflect the strong induction of numerous flagellar genes associated with the *lrhA*(Q278*) mutation present in ∆*tusA*-k.

To identify transcriptional regulators associated with the upregulated and downregulated genes, we performed regulon enrichment analysis (Table S2). When the top 10 enriched transcriptional regulators for the upregulated genes were ranked by FDR, only two were shared between Δ*tusA*-k and Δ*tusA*-t, indicating substantial differences in the regulatory networks of the two strains (Fig. 3C). Notably, the top-ranked regulators in Δ*tusA*-k included factors controlling flagellar biosynthesis and chemotaxis, such as FlhDC, FliA, and MotR, consistent with the enhanced flagellar gene expression observed in this strain. Taken together, these results suggest that the *lrhA*(Q278*) mutation in Δ*tusA*-k exerts a broad impact on global gene expression.

To clarify the effect of *tusA* deletion on gene expression, we performed gene ontology (GO) enrichment analysis of the upregulated and downregulated genes in Δ*tusA*-t. Among the upregulated genes, those involved in the tricarboxylic acid (TCA) cycle were the most significantly enriched (Fig. 3D). In the molecular function category, genes associated with the electron transport chain and transporters driven by ATP or redox potential were highly enriched (Fig. S3). In addition, the upregulated gene set included genes involved in RNA modification and RNA processing, which is consistent with the established role of TusA in tRNA thiolation (Fig. 3D). In contrast, the downregulated genes were primarily associated with diverse metabolic pathways and stress response functions (Fig. 3D). From the perspective of molecular function, redox-related activities were predominantly enriched, suggesting perturbations in Fe–S cluster-dependent proteins in the *tusA* knockout strain.

### Deletion of *tusA* confers resistance to cationic antimicrobial agents

**Fig 4:**
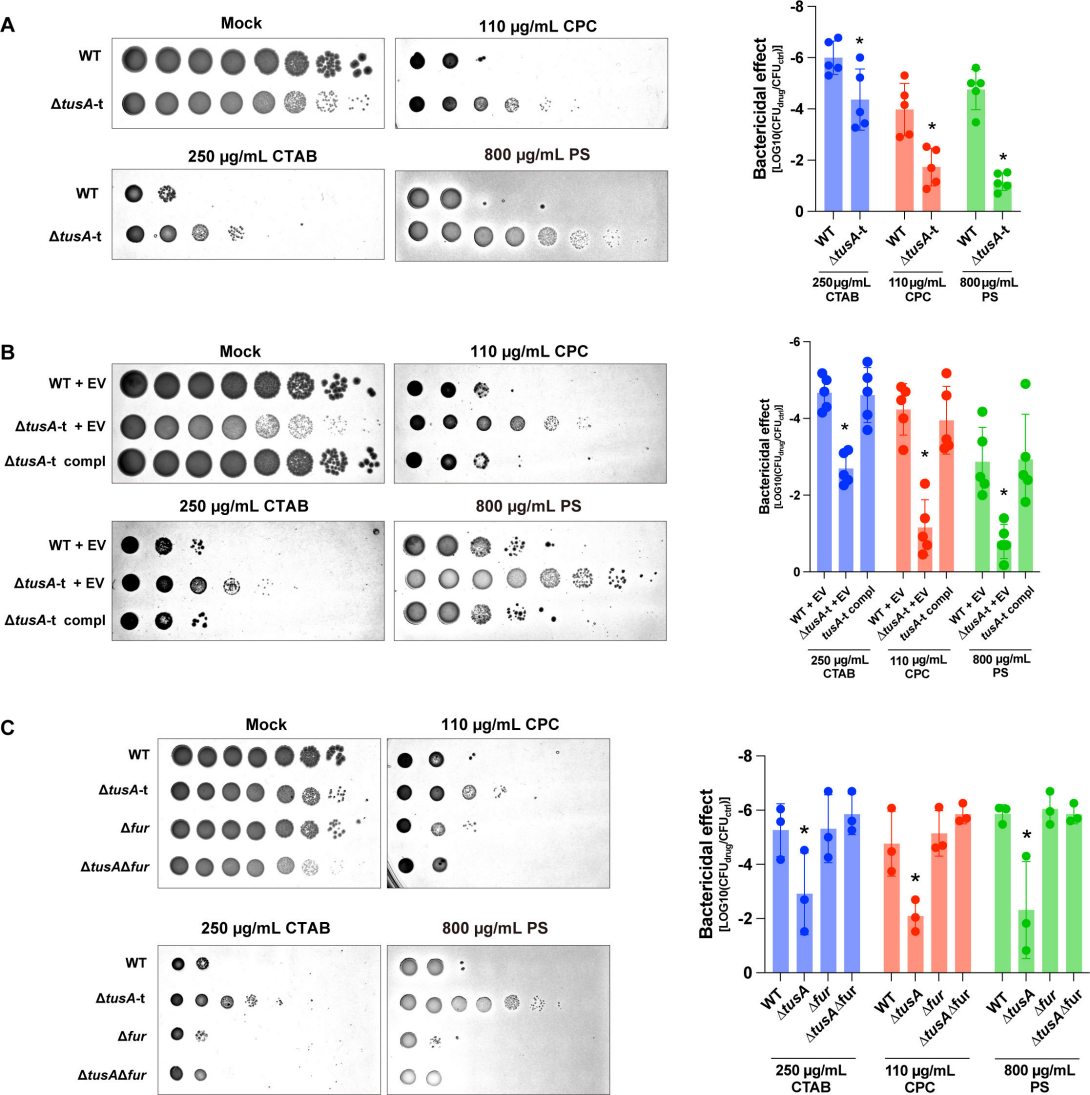
Δ*tusA*-t exhibits resistance to cationic antimicrobials. (**A**) Serial 10-fold dilutions of BW25113 (WT) and Δ*tusA*-t cultures were spotted onto LB agar or LB agar containing CTAB, CPC, or PS. (**B**) Serial 10-fold dilutions of cultures of WT and Δ*tusA*-t harboring pCA24N (WT + EV, Δ*tusA*-t + EV) or Δ*tusA*-t harboring pCA24N-tusA (Δ*tusA*-t compl) were spotted onto LB agar (mock) or LB agar containing CTAB, CPC, or PS. (**C**) Serial 10-fold dilutions of cultures of WT and deletion mutants (Δ*tusA*-t, Δ*fur*, and Δ*tusA* Δ*fur*) were spotted onto LB agar or LB agar containing CTAB, CPC, or PS. Bacterial survival was quantified as log_10_(CFU [drug]/CFU [control]). Data are presented as mean ± SD; Asterisks indicate significant differences from WT within the same medium (*n* = 3–5, *P* < 0.05, Tukey’s multiple comparisons test).

Antimicrobial susceptibility testing of Δ*tusA*-t revealed increased resistance to cationic compounds, including the quaternary ammonium detergents cetyltrimethylammonium bromide (CTAB) and cetylpyridinium chloride (CPC), as well as the polycationic peptide protamine sulfate (PS) (Fig. 4A). Complementation with a plasmid expressing *tusA* abolished the resistance to these cationic agents (Fig. 4B), indicating that the phenotype is attributable to the loss of *tusA*. We also examined the susceptibility of the wild-type strain to the anionic and non-ionic detergents sodium dodecyl sulfate (SDS) and Triton X-100, respectively. However, even at a high concentration (4%), the wild-type strain showed no detectable reduction in colony formation, precluding a reliable comparison of detergent susceptibility with Δ*tusA*-t (Fig. S4). Next, we evaluated the sensitivity of the Δ*tusA* Δ*fur* double mutant to cationic antimicrobial agents. The resistance observed in Δ*tusA*-t was abolished in the Δ*fur* background (Fig. 4C), indicating that the increased resistance of Δ*tusA*-t to cationic antimicrobial agents is mediated by Fur-dependent mechanisms.

**Fig 5:**
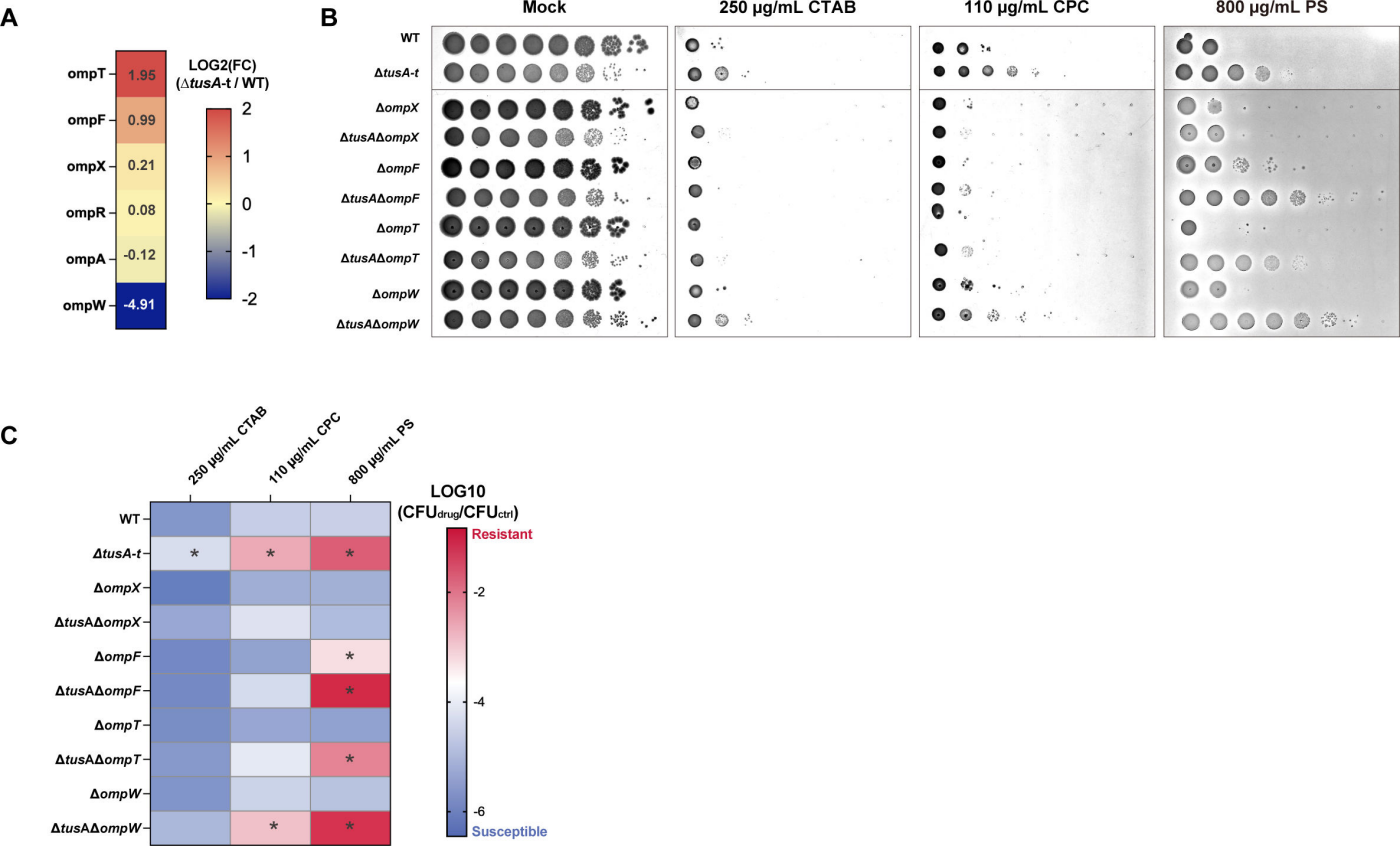
Cationic antimicrobial resistance in Δ*tusA*-t is dependent on *omp* genes. (**A**) Changes in *omp* gene expression in Δ*tusA*-t based on the RNA-seq data shown in Fig. 3. (**B**) Serial 10-fold dilutions of WT and deletion mutants (Δ*tusA*-t, Δ*ompX*, Δ*tusA* Δ*ompX*, Δ*ompF*, Δ*tusA* Δ*ompF*, Δ*ompT*, Δ*tusA* Δ*ompT*, Δ*ompW*, and Δ*tusA* Δ*ompW*) were spotted onto LB agar (mock) or LB agar containing CTAB, CPC, or PS. (**C**) Bacterial survival of Δ*tusA*-t and the indicated mutants under cationic antimicrobial treatment, quantified as log_10_(CFU [drug]/CFU [control]). Data are presented as a heat map (see Fig. S5 for the corresponding graph). Asterisks indicate significant differences compared with WT (*n* = 5, *P* < 0.05, Šídák’s multiple comparisons test).

Outer membrane protein (*omp*) genes are known to contribute to resistance to CTAB and protamine (22, 23), and several of these genes are transcriptionally regulated by Fur (24, 25). In Δ*tusA*-t, the expression of *ompT*, *ompF*, *ompX*, and *ompR* was increased, whereas *ompW* expression was decreased compared with the wild type (Fig. 5A). Based on these observations, we hypothesized that the resistance of Δ*tusA*-t to cationic antimicrobial agents is mediated by *omp* genes and tested this hypothesis using genetic analyses. The resistance of Δ*tusA*-t to CTAB was abolished in all tested *omp* deletion backgrounds (Fig. 5B and C; Fig. S3). For CPC, the resistance observed in Δ*tusA*-t was attenuated in all *omp* deletion mutants except Δ*ompW* (Fig. 5B and C; Fig. S3). In addition, the resistance of Δ*tusA*-t to protamine sulfate was abolished in the Δ*ompX* background. Collectively, these results indicate that the increased resistance of Δ*tusA*-t to cationic antimicrobial agents is mediated by altered expression of *omp* genes.

## DISCUSSION

In this study, we re-evaluated the physiological functions of *tusA* by identifying an unintended secondary mutation present in the Keio *tusA* knockout strain. Previous studies using this strain (11–13, 16) suggested that *tusA* exhibits pleiotropic effects; however, the presence of a secondary mutation that inactivates the transcription factor LrhA may have confounded the interpretation of the intrinsic functions of *tusA*.

In the comparison of gene expression between Δ*tusA*-t and Δ*tusA*-k, the overlap was lower for upregulated genes than for downregulated genes (Fig. 3A and B). This discrepancy is likely attributable to LrhA-mediated repression of FlhDC, the master transcriptional activator of flagellar genes. According to RegulonDB, FlhDC controls the expression of 93 genes (26), the majority of which are positively regulated. Consistently, marked upregulation of the flagellar gene *fliC* in Δ*tusA*-k has also been reported in previous studies (13), suggesting that the secondary mutation in *lrhA* may have influenced the outcomes of studies using Δ*tusA*-k. Although LrhA has been reported to regulate additional transcription factors, including LeuO and FimE, in addition to FlhDC (27, 28), no significant changes in the expression of these genes were observed in Δ*tusA*-k (Table S1). Taken together, these findings indicate that flagellar genes under the control of FlhDC are preferentially affected at the transcriptional level by the *lrhA* nonsense mutation in Δ*tusA*-k.

TusA has been reported to influence the translation of global transcriptional regulators, including *rpoS*, *fis*, and *fur*, through its role in mnm⁵s²U tRNA modification, thereby broadly affecting gene expression (11, 12). Meanwhile, LrhA has also been shown to negatively regulate *rpoS* translation in an Hfq-dependent manner (29). Hfq is an RNA chaperone that facilitates interactions between diverse small regulatory RNAs and their target mRNAs, thereby broadly modulating translation and mRNA stability across numerous genes (30). Notably, *fur* is also an Hfq target, and Hfq reduces *fur* mRNA stability and translation (31). Taken together, loss of LrhA function may substantially affect the translation of numerous genes through alterations in Hfq-mediated gene regulation. Therefore, previous studies using Δ*tusA*-k may need to be re-evaluated to determine the extent to which the observed phenotypes were influenced by the *lrhA* mutation.

**Fig 6:**
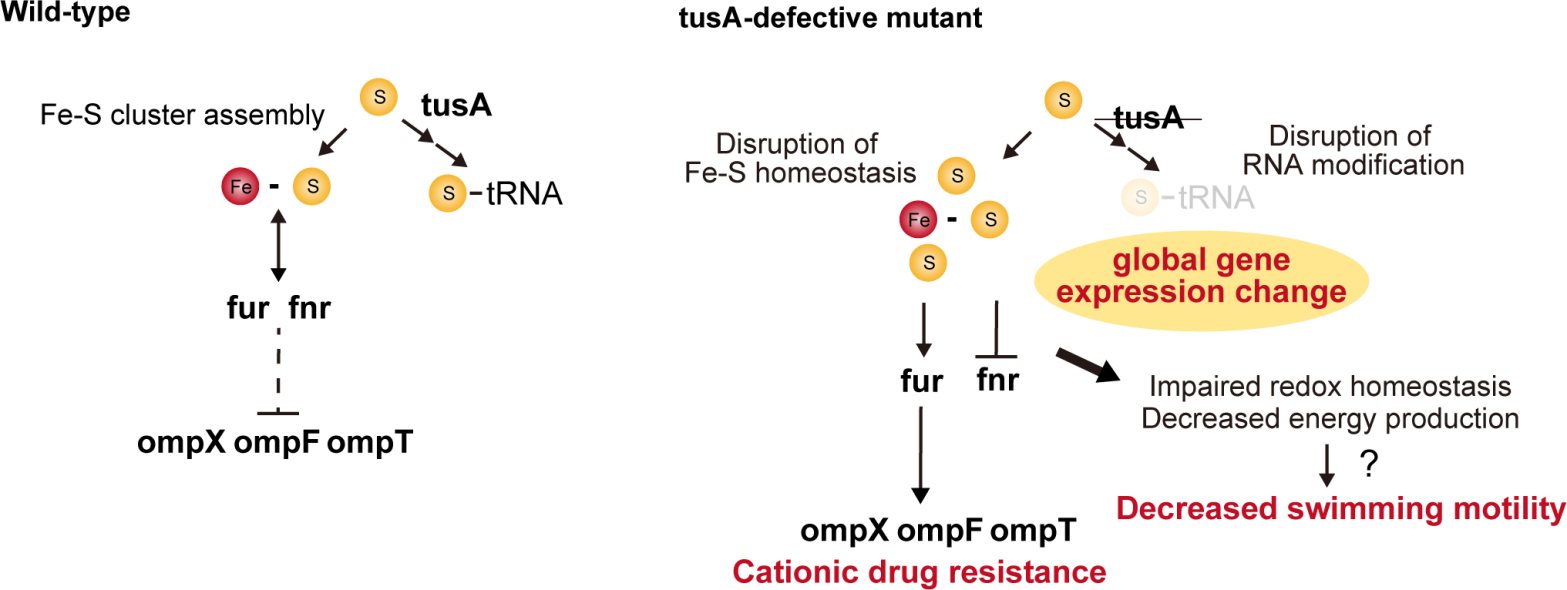
Proposed model in which a *tusA*-deficient mutant exhibits reduced swimming motility and resistance to cationic antimicrobials.

By performing RNA-seq analysis using a *tusA* knockout strain in which the secondary mutation was corrected, we clarified the direct effects of *tusA* deletion on global gene expression (Fig. 3). Based on these findings, we propose a model to explain the global transcriptional changes observed in the *tusA* mutant. Because TusA contributes to Fe–S homeostasis by allocating sulfur to tRNA thiolation (12), deletion of *tusA* is expected to disrupt intracellular Fe–S homeostasis (Fig. 6). Such disruption is likely to impair the function of Fe–S cluster-containing proteins. In particular, the activity of the Fe–S cluster-dependent transcription factor Fnr may be substantially affected. This notion is supported by the GO enrichment analysis, which revealed significant downregulation of genes involved in anaerobic respiration, oxidoreductase activity, and Fe–S cluster binding (Fig. 3D; Fig. S2), many of which belong to the Fnr regulon. The widespread repression of the Fnr regulon in the *tusA* knockout strain is also consistent with previous reports (13). Moreover, the cellular levels of [4Fe–4S] clusters are reduced in the *tusA* knockout mutant (12), which is in agreement with the fact that Fnr is active in its [4Fe–4S] form and inactive in its [2Fe–2S] form (32).

Many of the upregulated genes were involved in the TCA cycle. The transcription factor ArcA, which regulates these genes, modulates its activity in response to the redox state of quinones in the respiratory chain (Fig. 3D). In Δ*tusA*-t, expression of *cyoABCDE*, which encodes a quinol oxidase involved in quinone oxidation in the respiratory chain, was increased approximately 16-fold, whereas expression of *ndh*, which reduces quinones, was decreased to about one-seventh of the wild-type level (Table S1); both genes belong to the Fnr regulon (33). Inactivation of Fnr would therefore be expected to alter the expression of Fnr-regulated factors that control the redox state of the respiratory chain, thereby leading to changes in ArcA activity. Accordingly, impaired Fnr activity in the *tusA* knockout mutant may represent a central mechanism underlying the pleiotropic effects on gene expression and cellular physiology observed in this strain.

Δ*tusA*-t exhibited reduced swimming motility (Fig. 1A). Although deletion of *tusA* in the K-12 background also resulted in decreased swimming motility, no apparent reduction in flagellar formation was observed (Fig. 1B), suggesting that *tusA* deletion does not impair flagellar assembly. Furthermore, even in the Δ*rpoS* and Δ*fur* mutant backgrounds, in which swimming motility is enhanced through distinct mechanisms, deletion of *tusA* consistently led to reduced motility (Fig. S2). These findings suggest that the motility defect caused by *tusA* deletion is not attributable to impaired flagellar formation but rather to a more fundamental physiological alteration. One possible explanation is that disrupted redox homeostasis reduces the efficiency of aerobic energy production, thereby decreasing the power output of the flagellar motor (Fig. 6).

Deletion of *tusA* was suggested to confer resistance to cationic antimicrobial agents through Fur-dependent alterations in *omp* gene expression (Fig. 4 and 5). Resistance to PS appeared to be primarily mediated by *ompX*, whereas resistance to CTAB and CPC was influenced by the combined contributions of *ompX*, *ompF*, and *ompT*. Previous studies have reported that *ompF* and *ompW* are subject to Fur-dependent regulation (24, 25). In addition, intracellular free iron levels are elevated in the *tusA* knockout strain (12). Fur is a transcription factor that regulates iron homeostasis and is activated upon iron binding (34); thus, altered Fur activity in the *tusA* knockout strain may directly modulate the expression of multiple *omp* genes. Furthermore, changes in Fur activity may affect bacterial environmental responses and indirectly influence *omp* gene expression via the EnvZ/OmpR regulatory system (35, 36). Although the relationship between Fur and Fnr in the *tusA* deletion strain remains unclear, Fur has been proposed to interact with Fe–S cluster-associated pathways and to regulate the expression of the ISC and SUF systems (8), suggesting that altered Fur activity may also contribute to the reduced activity of Fnr.

*omp* genes encode outer membrane porins that mediate the permeation of various substances and contribute to drug resistance (37). OmpF is a highly expressed porin that typically forms channels allowing the diffusion of molecules ≤600 Da (38, 39). OmpF pores serve as entry pathways for various antibiotics, and loss of *ompF* reduces the cellular uptake of drugs such as β-lactams and quinolones, thereby conferring antibiotic resistance (40–42). Although the role of OmpX in substance permeation remains largely unclear, *E. coli* lacking *ompX* exhibits reduced virulence (43, 44) and increased resistance to SDS and hydrophobic antibiotics (45). Outer membrane porins primarily facilitate passive diffusion of molecules, and active export of drugs that have entered the cell is unlikely to be mediated by these proteins. In addition, cationic antimicrobial agents exert their antibacterial activity mainly by disrupting negatively charged bacterial membranes rather than by penetrating into the cytoplasm (46). Therefore, the resistance of the *tusA* knockout strain to cationic antimicrobial agents is unlikely to be due to altered permeability to these compounds. This interpretation is consistent with the observation that the sensitivity of Δ*ompF* and Δ*ompX* to cationic agents was comparable to that of the wild type (Fig. 5D and E). Omp proteins and lipopolysaccharides form heterogeneous domains in the outer membrane, and their spatial organization and network structure are key determinants of outer membrane properties (47). Thus, increased expression of *ompF* and *ompX* may alter outer membrane architecture, thereby enhancing resistance to cationic antimicrobial agents in the *tusA* knockout strain.

OmpT, unlike OmpX and OmpF, is not a porin but an outer membrane protease (48). A previous study demonstrated that OmpT exhibits proteolytic activity against PS, and a Δ*ompT* mutant shows increased sensitivity to protamine in liquid culture (23). In our study, although *ompT* expression was increased approximately fourfold in Δ*tusA*-t, OmpT did not appear to be the primary determinant of PS resistance (Fig. 5). The Δ*ompT* mutant exhibited only a slight increase in sensitivity to PS, and the magnitude of this effect was modest. This discrepancy may be attributable to differences in the mode of action of PS in liquid culture versus on solid agar. In contrast, OmpT appears to contribute to CTAB and CPC resistance in Δ*tusA*-t. OmpT has been reported to interact with membrane lipids and modulate membrane fluidity (49). Therefore, similar to OmpX and OmpF, altered *ompT* expression may modify outer membrane properties, thereby contributing to resistance to cationic antimicrobial agents in the *tusA* knockout strain.

## MATERIALS AND METHODS

### Bacteria and culture conditions

*Escherichia coli* K-12 strain, BW25113, and single-gene deletion mutants were obtained from the National BioResource Project, Japan (K-12, ME9012; BW25113, ME9062; Δ*tusA*-k, JW3435-KC; Δ*yfbQ*, JW2287-KC; Δ*fur*, JW0669-KC; Δ*rpoS*, JW5437-KC; Δ*ompX*, JW0799-KC; Δ*ompF*, JW0912-KC; Δ*ompT*, JW0554-KC; and Δ*ompW*, JW1248-KC). The *tusA* knockout mutant in which the secondary mutation was removed (Δ*tusA*-t) was constructed by bacteriophage P1*vir*-mediated transduction (50), using Δ*tusA*-k as the donor and BW25113 as the recipient. Except for the strains used in Fig. 2F and G, double gene knockout mutants were generated by P1*vir*-mediated transduction (50) using Δ*tusA*-k as the donor and Keio deletion mutants lacking the kanamycin resistance cassette (51) as the recipients. According to the genome sequence of BW25113 (GenBank accession number: CP122319), *tusA* is located at chromosomal positions 1,411,688–1,411,933, whereas *lrhA* is located at 208,754–209,692. The large chromosomal distance between these loci precludes co-transduction of the *lrhA* secondary mutation together with the *tusA* deletion by P1*vir*-mediated transduction. The strains used in Fig. 2F and G were constructed as described above, using BW25113 or kanamycin cassette-excised Δ*tusA*-t and Δ*tusA*-k strains as recipients and Δ*yfbQ* from the Keio collection as the donor. The *lrhA* locus in the resulting transductants was confirmed to be the wild-type allele by Sanger sequencing. All bacterial strains were grown on LB agar at 37°C. Single colonies were inoculated into 5 mL of LB medium (1% tryptone, 0.5% yeast extract, and 1% NaCl) and incubated at 37°C for 18 h with aerobic shaking in 50 mL sterile polypropylene conical tubes (Nunc; Thermo Fisher Scientific, USA).

### Plasmid construction and transformation

The plasmid carrying *tusA* (pCA24N-tusA) was isolated from JW3435-AM of the *Escherichia coli* ASKA clone library using a FastGene Plasmid Mini Kit (Nippon Genetics, Japan). DNA fragments containing *lrhA* or *lrhA*(Q278*), including the Shine–Dalgarno sequence, were amplified by PCR using genomic DNA from BW25113 and Δ*tusA*-k as templates and the oligonucleotide primers (5′-CTCGAATTCTCAGTATGAGCCGCCAGT-3′ and 5′-TGCAAGCTTTTACTCGATATCCCTTTCAAT-3′). The amplified fragments were digested with EcoRI and HindIII and ligated into the corresponding sites of the pMW118 vector, generating pMW118-lrhA and pMW118-lrhA(Q278*). The resulting plasmids were introduced into *E. coli* BW25113 and its gene deletion mutants by electroporation.

### Bacterial genome resequencing

Genomic DNA was extracted from overnight cultures of Δ*tusA*-k and Δ*tusA*-t using a QIAamp DNA Blood Mini Kit (Qiagen, Germany). The extracted genomic DNA was sequenced on a DNBSEQ-G400RS platform using paired-end sequencing (2 × 150 bp), generating approximately 0.5 Gb of total sequence data. The reads were mapped to the reference genome of BW25113 (GenBank accession number: CP122319) using CLC Genomics Workbench (version 11.0).

### RNA sequencing

Total RNA was extracted and sequenced as previously described (52). Briefly, 50 µL of overnight cultures of BW25113, Δ*tusA*-t, and Δ*tusA*-k were inoculated into 5 mL of LB medium and incubated aerobically at 37°C. When the cultures reached an OD_600_ of 0.7, 1.8 mL of each culture was immediately mixed with 200 µL of phenol in 5% ethanol by vortexing, chilled in ice water for 5 min, and centrifuged at 21,500 × *g* for 2 min. The cell pellets were frozen in liquid nitrogen and stored at −80°C for 2 h. The pellets were resuspended in 200 µL of lysis buffer (TE buffer containing 1% lysozyme and 1% SDS) and incubated at 65°C for 2 min. Total RNA was extracted using the RNeasy Mini Kit (Qiagen) according to the manufacturer’s instructions. rRNA was depleted using the NEBNext rRNA Depletion Kit, and cDNA libraries were prepared using either the TruSeq Standard Total RNA Kit (Illumina) or the MGIEasy Fast RNA Library Prep Set (MGI Tech, China). RNA sequencing was performed on a NovaSeq 6000 system (Illumina) or a DNBSEQ-G400RS platform (MGI Tech) to generate 100 or 150 bp paired-end reads, yielding at least 4 Gb of sequence data per sample. The reads were mapped to the *Escherichia coli* W3110 reference genome (GenBank accession number: NC_007779.1) using CLC Genomics Workbench (version 11.0). Differential gene expression analysis was performed using DESeq2 (53) in R (version 4.4.2) based on read count data for each gene. Gene symbols were assigned to locus tags using the Profiling of *E. coli* Chromosome database when annotations were not available in CLC Genomics Workbench. Adjusted *P* values and fold-change values were visualized using GraphPad Prism 9. GO enrichment analysis was conducted using ShinyGO (version 0.77) with *E. coli* MG1655 (Taxonomy ID: 511145) as the reference data set (54). Regulon enrichment analysis of differentially expressed genes was performed using RegulonDB (version 13.5.0) (26). The regulons of individual transcription factors were obtained from RegulonDB, and genes with a fold change ≥2 or ≤0.5 and an adjusted *P* value ≤0.05 were quantified using R (version 4.4.2) (55).

### Immunofluorescence

To visualize flagella, 100 µL of bacterial culture was placed onto a poly-L-lysine-coated coverslip and incubated for 1 h to allow cell attachment. The samples were washed three times with phosphate-buffered saline (PBS) and then incubated with 300 µL of anti-flagellin antibody (ab93713; Abcam, UK) diluted 1:4,000 in PBS at 37°C for 1 h. After three washes with PBS, 300 µL of Alexa Fluor 488-conjugated donkey anti-rabbit IgG (406416; BioLegend, CA, USA) diluted 1:4,000 in PBS was applied and incubated at 37°C for 40 min. The samples were then washed three times with PBS, and flagella were observed using an Axiovert 5 fluorescence microscope (Zeiss, Germany) equipped with a Plan-Apochromat 63×/1.4 oil immersion objective and an Axiocam 305 color camera. Flagellar frequency was quantified by particle analysis using Fiji software (version 2.14) (56) from triplicate images captured randomly under identical excitation intensity and magnification.

### Swimming assay

To assess swimming motility, 1 µL of an LB-grown bacterial culture (OD_600_ = 5) was inoculated into low-salt soft agar medium (1% tryptone, 0.25% NaCl, and 0.25% agar). Plates were incubated at 37°C for 19 or 24 h, after which the swimming halos were photographed. The halo diameters (Feret’s diameter) were measured using Fiji software (version 2.14) (56).

### Assessment of cationic antimicrobial resistance

CTAB, CPC, and PS were dissolved in ultrapure water at stock concentrations of 50, 32.5, and 20 mg/mL, respectively, and added to autoclaved LB agar to achieve the indicated final concentrations. SDS and Triton X-100 were dissolved in ultrapure water at 8% (wt/vol) and mixed with an equal volume of 2× concentrated autoclaved LB agar before plate preparation. LB-grown *E. coli* cultures were serially diluted 10-fold in LB broth using a 96-well microplate. Aliquots of the diluted cultures (5 µL for LB control, CTAB, CPC, and PS; 3 µL for SDS and Triton X-100) were spotted onto the agar plates using a multichannel micropipette. The plates were incubated at 37°C for 18 h and photographed using a digital camera.

## SUPPLEMENTAL MATERIAL

Supplemental figures jb.00103-26-s0001.pdf Figures S1 to S5.

Table S1 jb.00103-26-s0002.xlsx RNA-seq data for Δ*tusA*-k and Δ*tusA*-t.

Table S2 jb.00103-26-s0003.xlsx Regulon enrichment analysis of Δ*tusA*-k and Δ*tusA*-t.

ASM does not own the copyrights to Supplemental Material that may be linked to, or accessed through, an article. The authors have granted ASM a non-exclusive, world-wide license to publish the Supplemental Material files. Please contact the corresponding author directly for reuse.
