# Supplementary material for: Knockout of tusA confers cationic antimicrobial resistance via fur and omp genes in Escherichia coli
Source: J Bacteriol. 2026 Apr 22;208(5):e00103-26. doi: 10.1128/jb.00103-26 (PMC13192261; doi:10.1128/jb.00103-26)
Supplement: Supplemental figures — Figures S1 to S5. [file jb.00103-26-s0001.pdf]

Supplementary Figures

Fig. S1. *tusA* complementation restores the growth of Δ*tusA*-k and Δ*tusA*-t in LB medium.

Growth curves of BW25113, Δ*tusA*-k, and Δ*tusA*-t harboring pCA24N (WT + pCA24N, Δ*tusA*-k + pCA24N, and Δ*tusA*-t + pCA24N) or pCA24N-tusA (Δ*tusA*-k + pCA24N-tusA and Δ*tusA*-t + pCA24N-tusA) in LB medium at 37 °C.

Fig. S2. Reduced swimming motility caused by *tusA* deletion is also observed in *fur*- or *rpoS*-deficient backgrounds.

Swimming motility of BW25113 (wild type [WT]), Δ*tusA*-t, Δ*fur*, Δ*tusA* Δ*fur*, Δ*rpoS*, and Δ*tusA* Δ*rpoS*. Soft agar plates were incubated at 37 °C for 19 h. Scale bar, 10 mm. Quantification of swimming halo diameters is shown. The data for WT and Δ*tusA*-t are the same as those in Fig. 1A. Data are presented as mean ± SD; different letters indicate significant differences (n = 3–4, $P < 0.01$, Tukey's multiple comparisons test).

Fig. S3. GO enrichment analysis of molecular function categories for differentially expressed genes in Δ*tusA*-t.

Fig. S4. Assessment of resistance of ΔtusA-t to anionic and non-ionic detergents.

Serial 10-fold dilutions of WT and Δ*tusA*-t cultures were spotted onto LB agar or LB agar containing sodium dodecyl sulfate (SDS) or Triton X-100.

Fig. S5. Quantitative analysis of the data shown in Fig. 5B.

Bacterial survival of Δ*tusA*-t and the indicated mutants (Δ*ompX*, Δ*tusA* Δ*ompX*, Δ*ompF*, Δ*tusA* Δ*ompF*, Δ*ompT*, Δ*tusA* Δ*ompT*, Δ*ompW*, and Δ*tusA* Δ*ompW*) was quantified as $\log_{10}$[CFU (drug)/CFU (control)]. Data are presented as mean ± SD; asterisks indicate significant differences within the same medium (n = 5, $P < 0.05$, Šídák's multiple comparisons test).

Figure S1

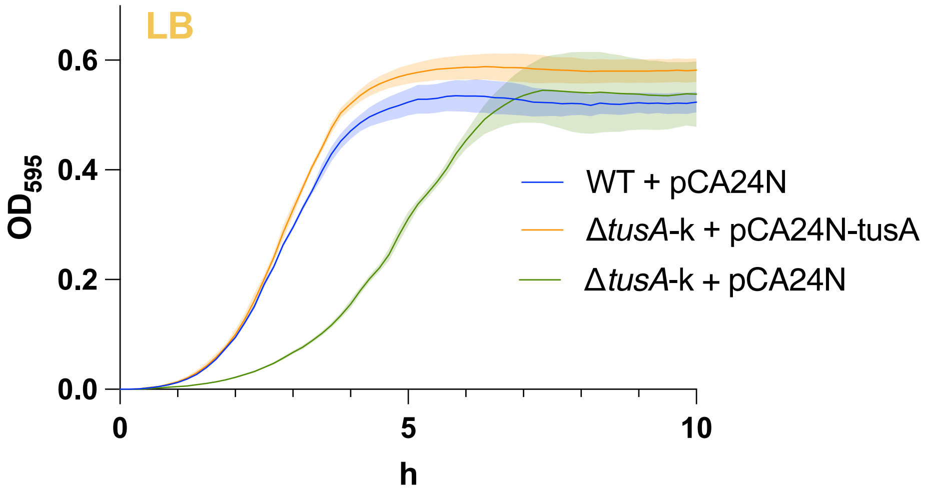
LB
0.6
0.4
0.2
0.0
OD_{595}
0
5
10
h
WT + pCA24N
ΔtusA-k + pCA24N-tusA
ΔtusA-k + pCA24N

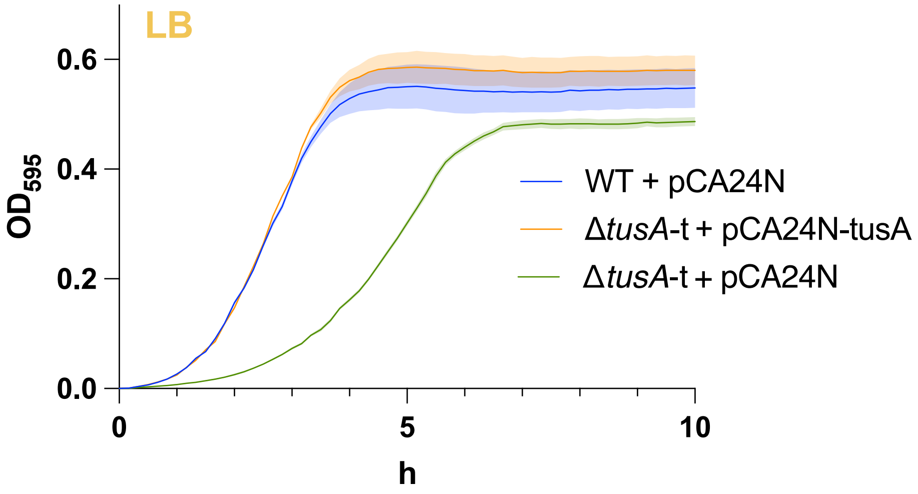
LB
0.6
0.4
0.2
0.0
OD_{595}
0
5
10
h
WT + pCA24N
ΔtusA-t + pCA24N-tusA
ΔtusA-t + pCA24N

Figure S2

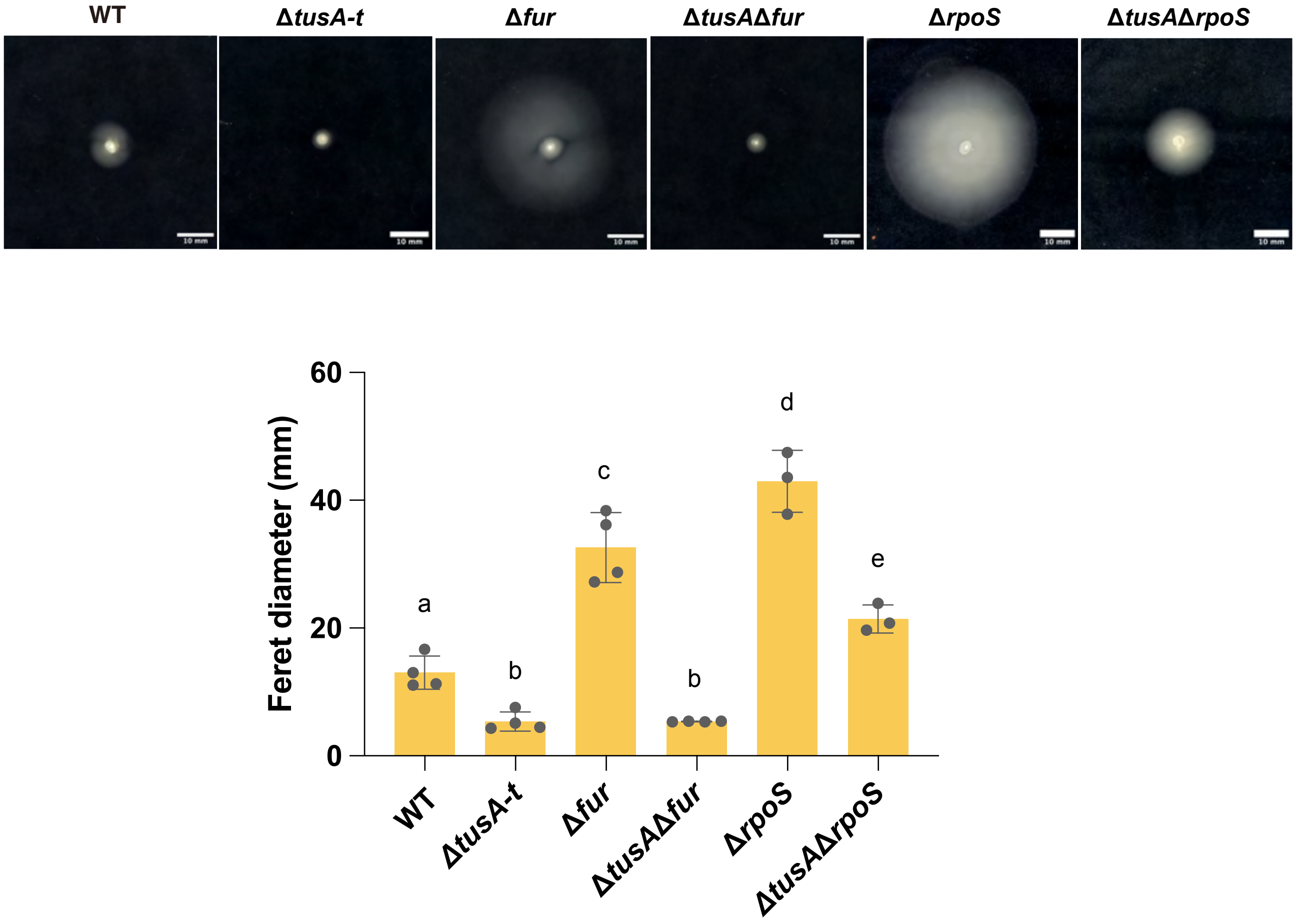
WT
ΔtusA-t
Δfur
ΔtusAΔfur
ΔrpoS
ΔtusAΔrpoS
Feret diameter (mm)
60
40
20
0
a
b
c
b
d
e
WT
ΔtusA-t
Δfur
ΔtusAΔfur
ΔrpoS
ΔtusAΔrpoS

Figure S3

Up genes (Molecular function)

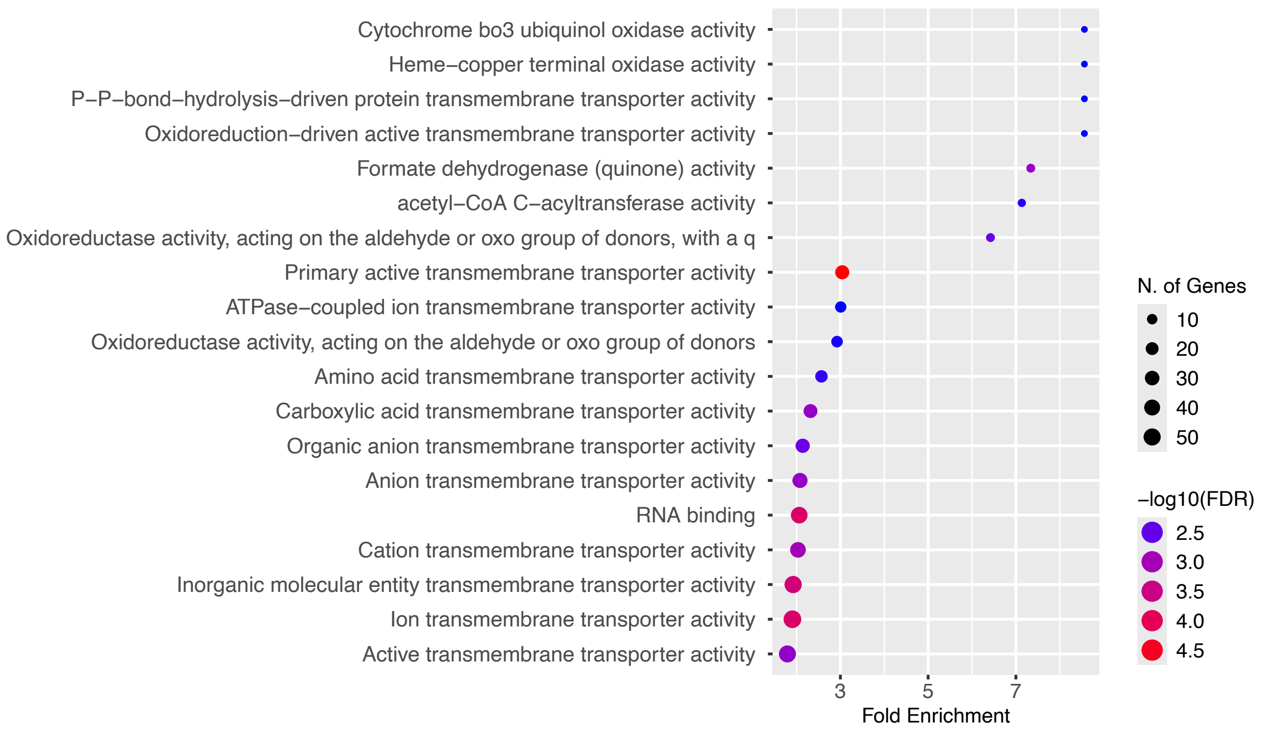

Down genes (Molecular function)

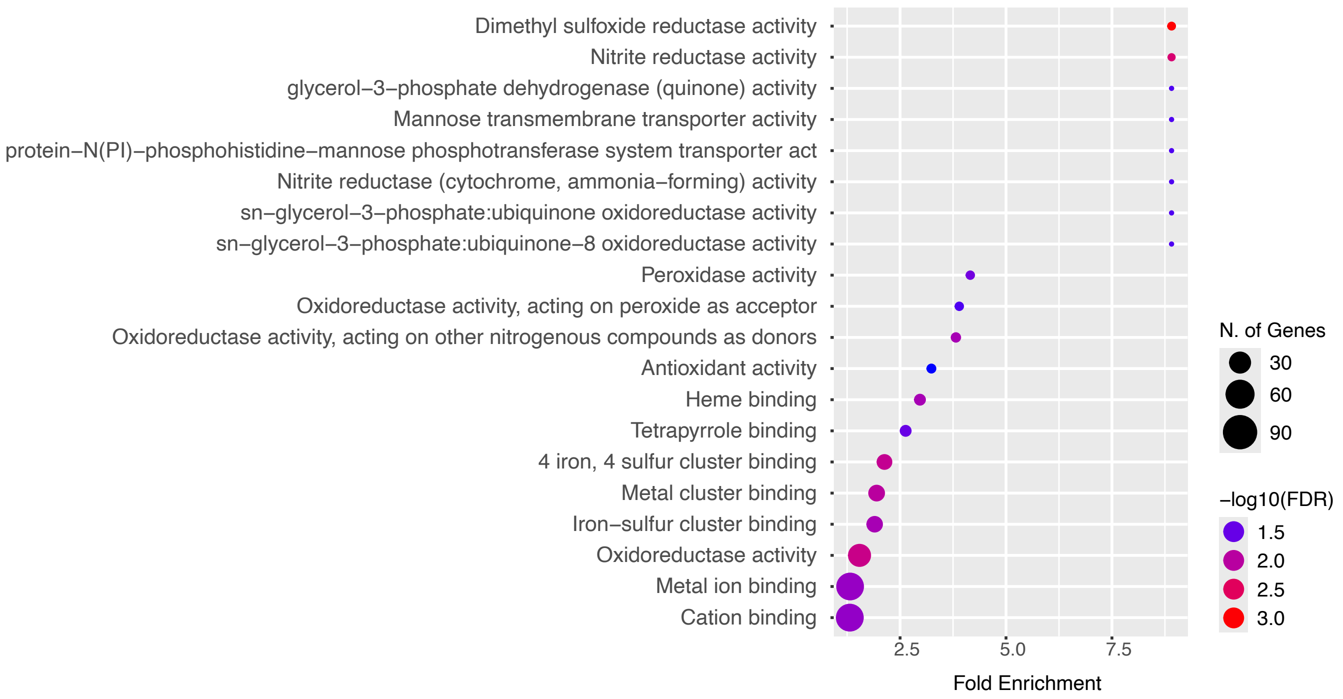

Figure S4

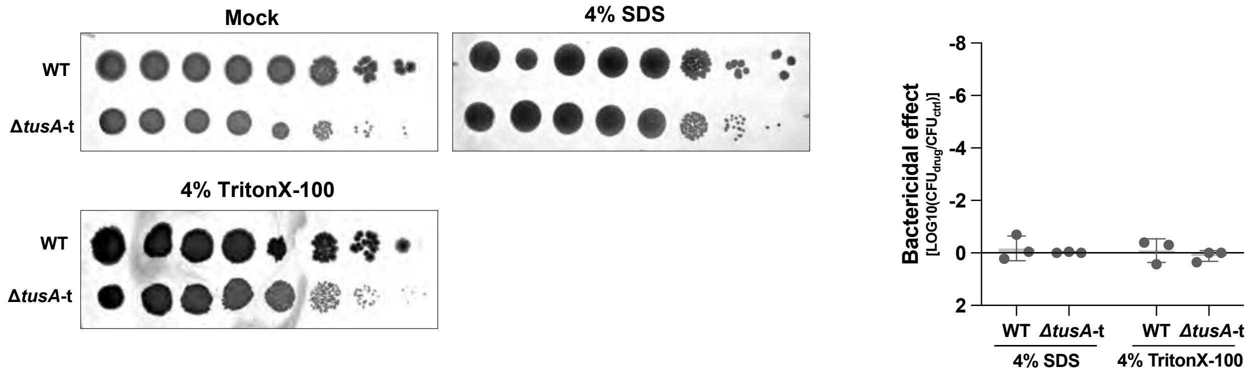

Mock
4% SDS
WT
ΔtusA-t
4% TritonX-100
WT
ΔtusA-t
Bactericidal effect
[LOG10(CFUdrug/CFUctrl)]
-8
-6
-4
-2
0
2
WT
ΔtusA-t
WT
ΔtusA-t
4% SDS
4% TritonX-100

Figure S5

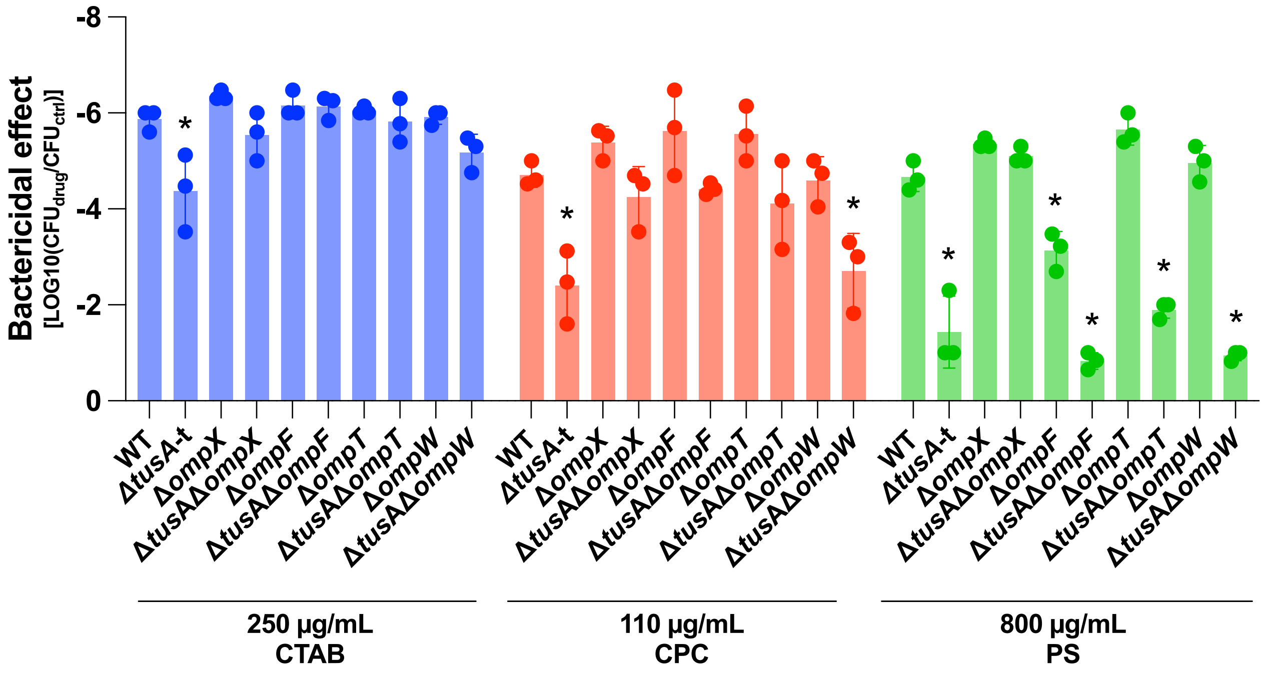
Bactericidal effect
[LOG10(CFUdrug/CFUctrl)]
-8
-6
-4
-2
0
WT
ΔtusA-t
ΔompX
ΔtusAΔompX
ΔompF
ΔtusAΔompF
ΔompT
ΔtusAΔompT
ΔompW
ΔtusAΔompW
250 µg/mL
CTAB
110 µg/mL
CPC
800 µg/mL
PS
